# A scoping review of factors associated with antimicrobial-resistant *Campylobacter* species infections in humans

**DOI:** 10.1017/S0950268823000742

**Published:** 2023-06-07

**Authors:** Christine M. Neustaedter, Kelsey Robertson, Dana Tschritter, Richard J. Reid-Smith, Melissa C. MacKinnon, Colleen P. Murphy, Brennan Chapman, Norman F. Neumann, Simon J. G. Otto

**Affiliations:** 1 School of Public Health, University of Alberta, Edmonton, AB, Canada; 2 HEAT-AMR Research Group, School of Public Health, University of Alberta, Edmonton, AB, Canada; 3 Antimicrobial Resistance – One Health Consortium, University of Calgary, Calgary, AB, Canada; 4 Food-Borne Disease and Antimicrobial Resistance Surveillance Division, Centre for Food-Borne, Environmental, and Zoonotic Infectious Diseases, Public Health Agency of Canada, Guelph, ON, Canada; 5 Department of Population Medicine, Ontario Veterinary College, University of Guelph, Guelph, ON, Canada; 6 Healthy Environments Thematic Area Lead, Centre for Healthy Communities, School of Public Health, University of Alberta, Edmonton, AB, Canada

**Keywords:** Antimicrobial resistance, antimicrobial drugs, *Campylobacter*, food-borne infections, risk factor

## Abstract

Human infection with antimicrobial-resistant *Campylobacter* species is an important public health concern due to the potentially increased severity of illness and risk of death. Our objective was to synthesise the knowledge of factors associated with human infections with antimicrobial-resistant strains of *Campylobacter.* This scoping review followed systematic methods, including a protocol developed *a priori.* Comprehensive literature searches were developed in consultation with a research librarian and performed in five primary and three grey literature databases. Criteria for inclusion were analytical and English-language publications investigating human infections with an antimicrobial-resistant (macrolides, tetracyclines, fluoroquinolones, and/or quinolones) *Campylobacter* that reported factors potentially linked with the infection. The primary and secondary screening were completed by two independent reviewers using Distiller SR®. The search identified 8,527 unique articles and included 27 articles in the review. Factors were broadly categorised into animal contact, prior antimicrobial use, participant characteristics, food consumption and handling, travel, underlying health conditions, and water consumption/exposure. Important factors linked to an increased risk of infection with a fluoroquinolone-resistant strain included foreign travel and prior antimicrobial use. Identifying consistent risk factors was challenging due to the heterogeneity of results, inconsistent analysis, and the lack of data in low- and middle-income countries, highlighting the need for future research.

## Introduction

*Campylobacter* species is one of the leading causes of acute diarrheic illness, accounting for 16% of foodborne illnesses globally [1] and 8.42% of foodborne illnesses in Canada [2]. Infections are characterised by acute, watery diarrhoea progressing to bloody diarrhoea and often accompanied by abdominal pain, but vomiting is uncommon [3]. *Campylobacter* infection has an incubation period of 2–4 days and most people recover within 2–5 days [4]. An uncomplicated infection typically only requires supportive care to avoid dehydration [4]; however, some cases develop bacteraemia [5]. Although uncommon, complications related to *Campylobacter* infections include but are not limited to reactive arthritis, irritable bowel syndrome, Guillain–Barré syndrome (GBS), and Miller Fisher Syndrome, a variant of GBS, which are autoimmune disorders characterised by nerve damage, muscle weakness, and sometimes paralysis [5, 6].

Fluoroquinolone and macrolide antimicrobials can be used in the treatment of complicated *Campylobacter* infections to reduce the duration of illness [7]. There is evidence that inappropriate antimicrobial prescribing practices occur in Canada for *Campylobacter* infections, such as prescribing antimicrobials after symptoms have resolved, before the culture results have confirmed the diagnosis of *Campylobacter*, or treatment before the collection of a sample [8]. Furthermore, antimicrobials not suggested by clinical antimicrobial stewardship guidelines have also been prescribed [9]. Human infections with strains resistant to macrolides, fluoroquinolones/quinolones, and other antimicrobial classes including tetracyclines occur [10], and these infections may have an increased risk of an adverse health event such as a longer duration of illness, hospitalisation, invasive illness, or death, than patients with a susceptible infection [11–13].

There is a large amount of research on factors associated with human *Campylobacter* infections, including undercooked meat, especially chicken, contaminated unpasteurised milk, animal contact, and contaminated water [4]. However, despite this wealth of research, searches on 21 January 2020, in Ovid Medline®, Cochrane Library, Joanna Briggs Institute (JBI) Systematic Review Registry, and Google Scholar did not identify any scoping or systematic reviews on factors associated with infections with antimicrobial-resistant *Campylobacter.* The objective of this scoping review was to synthesise the published literature on factors associated with human infections with antimicrobial-resistant strains of *Campylobacter* species, with a focus on resistance to macrolides, tetracyclines, fluoroquinolones, and/or quinolones.

## Methods

### Protocol, search, and information sources

The review followed the systematic search methods outlined in the JBI Reviewer’s Manual [14] and is reported according to the PRISMA Scoping Review reporting guidelines [15]. The protocol was registered with the JBI Systematic Review Register on 5 February 2020, and is available in the Supplementary Material (S1). The PRISMA-Scoping Review checklist is provided in Supplementary Table S1.

A comprehensive search strategy was developed in consultation with a librarian to identify articles that studied human infections with antimicrobial-resistant *Campylobacter.* An example search string for MEDLINE® in Ovid® is shown in Supplementary Table S2. The complete search strings (S1) were used to search MEDLINE®, AGRICOLA™ in ProQuest®, Centre for Agriculture and Bioscience abstracts in Web of Science, EMBASE® in Ovid, and Scopus®. Grey literature sources included the World Health Organization’s Global Index Medicus, the Bielefield Academic Search Engine, and the first 250 results from Google Scholar when sorted by relevance. The search was completed on 5 February 2020, and was updated on 7 May 2021. Articles were de-duplicated in three stages in Mendeley (Version 1.19.8, Elsevier, Amsterdam, Netherlands), EndNote (Version X9.2, Clarivate Analytics, London, United Kingdom), and DistillerSR (Version 2.35, Evidence Partners, Ottawa, ON, Canada).

### Eligibility criteria

To be included, articles, theses, and dissertations had to be an analytic study that used a comparison group and reported on factors potentially associated with human infections with a strain of *Campylobacter* resistant to an antimicrobial of interest: macrolides, tetracyclines, and/or fluoroquinolones/quinolones (collectively referred to as fluoroquinolones hereafter). Resistance had to be determined by recognised laboratory antimicrobial susceptibility testing methods such as disc diffusion or broth micro-dilution. Review articles, commentaries, opinion pieces, editorials, newspaper articles, books, book chapters, and conference proceedings were excluded. No limits were applied to language, geographical location, *Campylobacter* species, or the date of publication. Non-English articles identified during screening were excluded. Included studies had to report human *Campylobacter* infections confirmed by recognised laboratory methods. Studies on nonhuman research, infections other than *Campylobacter*, colonisation instead of infection, or that failed to confirm a *Campylobacter* infection by recognised laboratory methods were excluded.

Factors associated with human infections with a resistant strain of *Campylobacter* were defined as observations that were measured and quantified, with the potential for identifying a reported statistical relationship to antimicrobial resistance (AMR) [16], which included but were not limited to age, recent travel, or pre-existing medical conditions. The comparator group had to be appropriate for the study design. For example, the comparator group for case–control studies were infections with strains of *Campylobacter* that were susceptible to the antimicrobials of interest. Inherently, the comparator group had to be *Campylobacter* isolates from human infections that were susceptible to the antimicrobials of interest, to compare to the resistant isolates from human infections.

Articles were screened for eligibility via a two-stage screening process by two independent reviewers. Article titles, abstracts, and keywords were screened in the first stage, and articles proceeded to secondary screening if both reviewers determined all eligibility criteria were met or unclear (S1). Secondary full-text screening by both reviewers included articles that answered yes to all eligibility criteria. The reasons for exclusion were documented. Reviewers resolved conflicts through discussion.

### Data collection and synthesis

Data regarding authorship, publication date, the location of study, study type (defined by the authors or assigned by the reviewers), AMR outcome(s), *Campylobacter* species, the site of infection, factor description and descriptive data, results of measures of association (if considered), and the type of analysis (univariable vs. multivariable where reported) were extracted by one reviewer in Distiller SR® and analysed in Excel® (Microsoft, Redmond, WA) and using the R Metaphor package (v4.1.1, R Core Team, 2021). Tables and figures present key findings in the results, whereas the Supplementary Material provides comprehensive results from the study. Factors were combined into themed categories for comparison. For relative associations, an odds ratio (OR) with a value of less than 1 is generally interpreted as a protective factor, whereas a value of greater than 1 was interpreted as a risk factor, meaning that either was associated with a decreased or increased risk of infection with a resistant strain of *Campylobacter*, respectively.

## Results

### Selection of information sources

**Figure 1.**
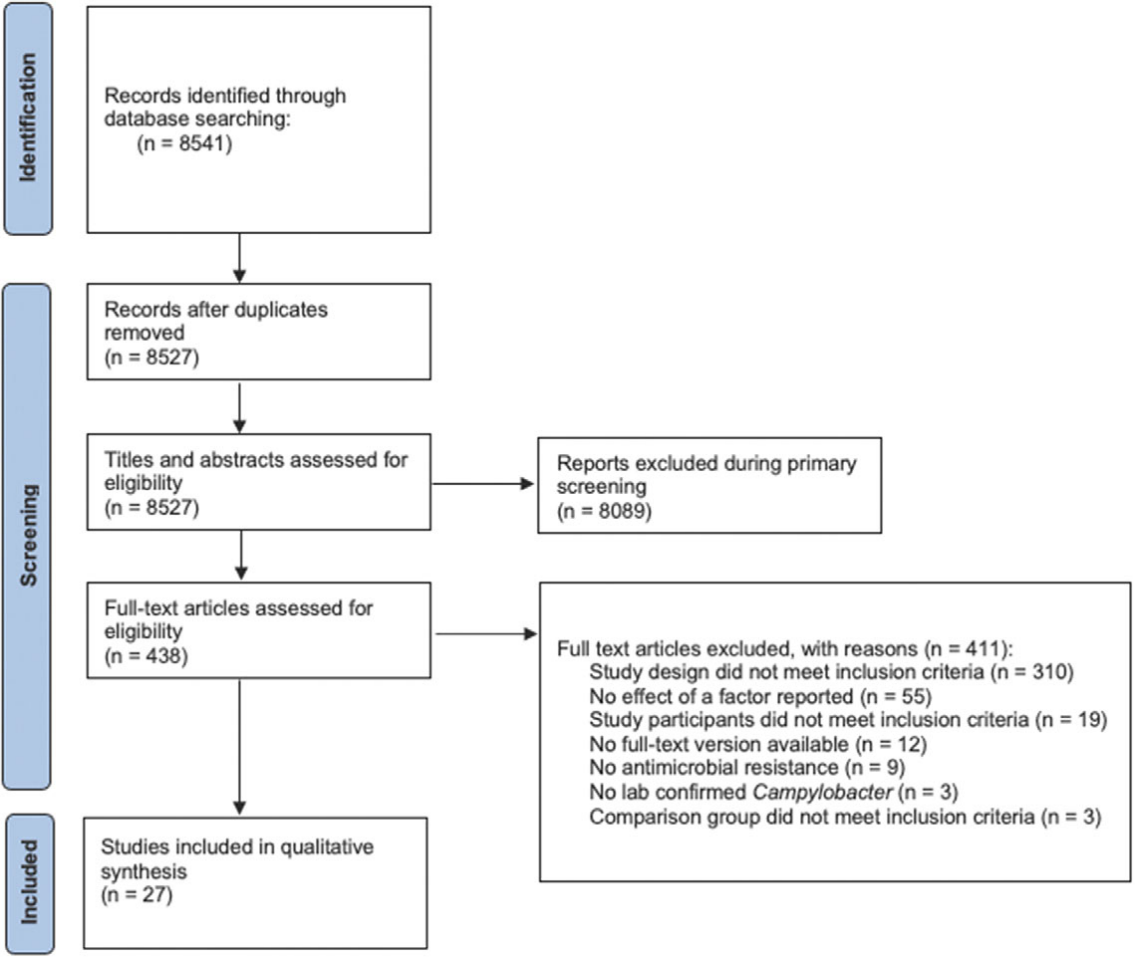
PRISMA scoping review flow diagram of the study selection process for the scoping review of human infections with an antimicrobial-resistant strain of *Campylobacter* species.

Our search identified 8,527 unique articles. Primary and secondary screening excluded 8,089 and 411 articles, respectively, including 12 where we could not locate a full-text document after additional inquiry through library requests (Figure 1). The review included 27 articles that met all inclusion criteria.

### Characteristics of information sources

The characteristics of included articles (*n* = 27) are included in Table 1. Complete extracted data for all studies are included in Supplementary Table S3. All articles were published between 1998 and 2018 except for one in 1988. The most common countries included the United States (*n* = 6), Denmark (*n* = 4), Canada (*n* = 3), and the United Kingdom (*n* = 3). Study designs included cross-sectional (*n* = 16), case–control (*n* = 4), case–case–control (*n* = 1), and various cohort designs (*n* = 6). The most commonly reported age range of participants was 20–50 years, but variations in reporting details made summarising age characteristics difficult. Fourteen studies reported the gender or sex of participants, but rarely included it in the analysis, whereas the rest did not report (*n* = 9) or did not include females in their study (*n* = 4). Most articles studied gastrointestinal infections (*n* = 19), and the most common species included was *Campylobacter jejuni* (*n* = 22). Six studies reported results for multivariable analyses, whereas the remaining 21 only reported results from univariable analyses if at all. Often, studies reported resistance to different antimicrobials. The most reported factor results were for resistance to fluoroquinolones (*n* = 20) and quinolones (*n* = 9), while resistance to macrolides (*n* = 13) and tetracyclines (*n* = 7) were also considered.

**Table 1.** Key characteristics of peer-reviewed references included in the scoping review of factors related to human infections with an antimicrobial-resistant strain of *Campylobacter* species

Study design^a^	Location	AMR	Species	Infection site^b^	Total sample size	Age details (years)^c^	Percentage of female^d^	Author and year
*Case–control (n = 4)*								
	Denmark	Quinolones Fluoroquinolones	*jejuni*	NS	126	Mean = 33 IQR = 20–45	83.3%	Engberg et al. (2004) [12]
	India	Macrolides Quinolones Fluoroquinolones Tetracyclines	*jejuni*	GI	400	Mean (cases) = 37 Mean (cont.) = 39.3	41.8%	Kownhar et al. (2007) [36]
	United Kingdom	Fluoroquinolones	NS	NS	556	Med (cases) = 53 Med (comp.) = 49	50.7%	Evans et al. (2009) [17]
	United States	Quinolone	*jejuni*	GI	390	NS	NS	Smith et al. (1999) [24]
*Case–case–control (n = 1)*								
	France	Fluoroquinolones	*jejuni*, *coli*, *fetus*, *lari*	GI	570	Mean (cases) = 19.5 Mean (cont.) = 20	0%	Gallay et al. (2007) [20]
*Cross-sectional (n = 16)*								
	Australia	Macrolides Quinolones Fluoroquinolones Tetracyclines	*jejuni*	GI	155	NS	NS	Sharma et al. (2003) [25]
	Australia	Fluoroquinolones	*upsaliensis*	GI	20	Mean = 40 Range = 27–53	10%	Jenkin et al. (1998) [35]
	Bosnia and Herzegovina	Macrolides Fluoroquinolones	*jejuni*, *coli*	GI	2,491	Med. range = 0–6 Range = 0–64+	NS	Uzunovic-Kamberovic et al. (2009) [41]
	Canada	Fluoroquinolones	*jejuni*, *coli*, NS	NS	210	16+	45.2%	Johnson et al. (2008) [21]
	Denmark	Macrolides Fluoroquinolones	NS	GI	10,475	Range = 0–80+	NS	Koningstein et al. (2011) [39]
	Denmark	Macrolides Quinolones Tetracyclines	*jejuni*	NS	1,023	NS	NS	Skjot-Rasmussen et al. (2009) [32]
	Finland	Fluoroquinolones	*jejuni*	GI	166	NS	59.6%	Feodoroff et al. (2010) [27]
	Finland	Fluoroquinolones	*Jejuni*	GI	354	NS	NS	Hakanen et al. (2003) [29]
	Ireland	Fluoroquinolones Tetracycline	15 *jejuni*, 2 *coli*	GI	15	Mean = 29.4 Range = 1–67	26.7%	Moore et al. (2002) [40]
	Netherlands	Macrolides Fluoroquinolones Tetracyclines	94% *jejuni*	NS	18,856	NS	NS	van Hees et al. (2007) [33]
	United Kingdom	Macrolides Fluoroquinolones	*jejuni*	GI	174	Med. = 2 Range = <1–75	40.2%	Ghunaim et al. (2015) [28]
	United Kingdom	Fluoroquinolones	*jejuni*	GI	495	Mean (cases) = 39 Mean (cont.) = 38	52.3%	CSSSC^e^ et al. (2002) [18]
	United States	Macrolides Quinolones	*jejuni*	GI/BS	16,549	Med. = 38	45.0%	Patrick et al. (2018) [31]
	United States	Fluoroquinolones	*jejuni*	NS	94	Med. = 23.5 Range = <2–50+	42.9%	Cha et al. (2016) [19]
	United States	Macrolides Quinolones	Mostly *jejuni*	GI	24,433	Mean (cases) = 37.1 Mean (comp.) = 36.2	45.5%	Ricotta et al. (2014) [34]
	United States	Fluoroquinolones	NS	GI	740	Med. = 34 Range = <1–96	46.0%	Nelson et al. (2004) [30]
*Cohort (n = 2)*								
	Denmark	Macrolides Quinolones	NS	GI	3,541	Mean = 27.4 Range = 0.2–92.3	NS	Helms et al. (2005) [11]
	United States	Macrolides	*jejuni*, *coli*	GI	4	Mean = 47 Range = 39–67	0%	Perlman et al. (1988) [23]
*Prospective cohort (n = 1)*								
	Belgium	Fluoroquinolones	*jejuni*	GI	1,730	Mean = 33 Range = <1–73	NS	Bottieau et al. (2011) [26]
*Retrospective cohort (n = 3)*								
	Canada	Quinolones Fluoroquinolones Tetracyclines	*jejuni*	GI	31	Range = 21–64	0%	Gaudreau et al. (2015) [38]
	Canada	Macrolides Fluoroquinolones Tetracyclines	*jejuni*	GI	14	Range = 26–40	0%	Gaudreau et al. (2003) [37]
	Taiwan	Macrolides	*jejuni*, *coli*	BS	21	Med. = 45 Range = 4–81	42.9%	Lu et al. (2000) [22]

a When a study design was not specified by the authors, the study design was determined by the first author during data extraction based on the reported methods.

b Infection type specified or determined during data extraction where possible; BS, blood-stream infection; GI, gastrointestinal infection; NS, not specified/could not be determined.

c Specified or calculated during data extraction where possible; comp., comparisons; cont., controls; IQR, interquartile range; Med., median; NS, not specified.

d Percentage of female versus other, specified in the article or calculated during the data extraction where possible; NS, not specified.

e CSSSC, *Campylobacter* Sentinel Surveillance Scheme Collaborators.

### Information about factors

**Figure 2.**
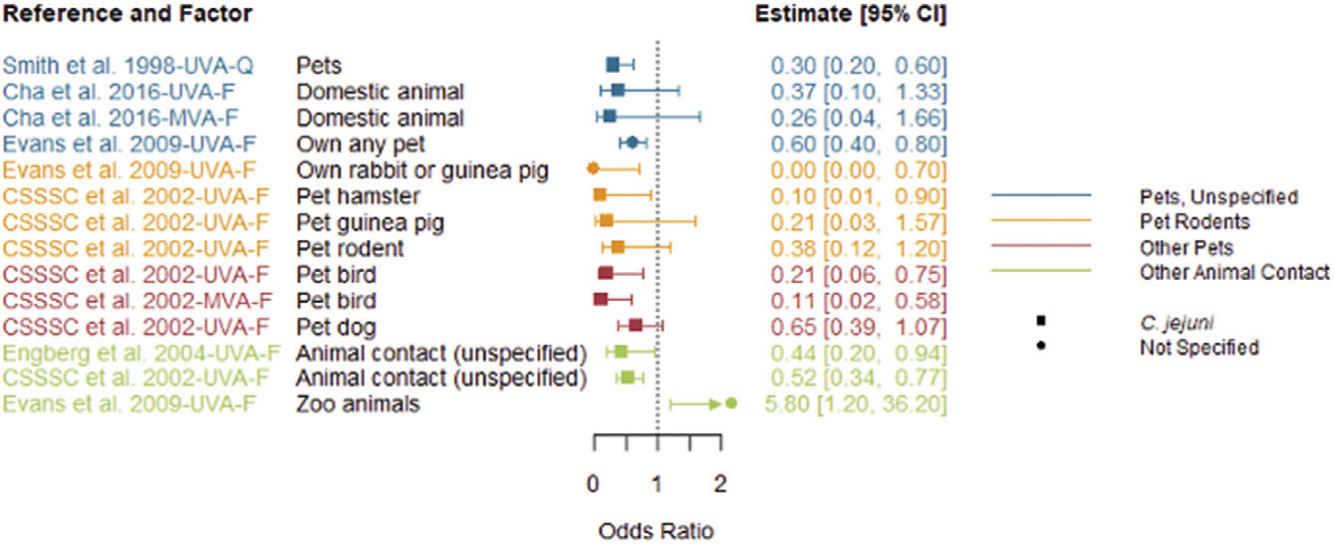
Animal contact factors identified in studies included in the scoping review for human infections with antimicrobial-resistant *Campylobacter* strains compared to infection with susceptible strains, limited to studies reporting odds ratios. *Note*: CSSSC, *Campylobacter* Sentinel Surveillance Scheme Collaborators; *F*, fluoroquinolone-resistant outcome; MVA, multivariable analysis result; *Q*, quinolone-resistant outcome; UVA, univariable analysis result

**Figure 3.**
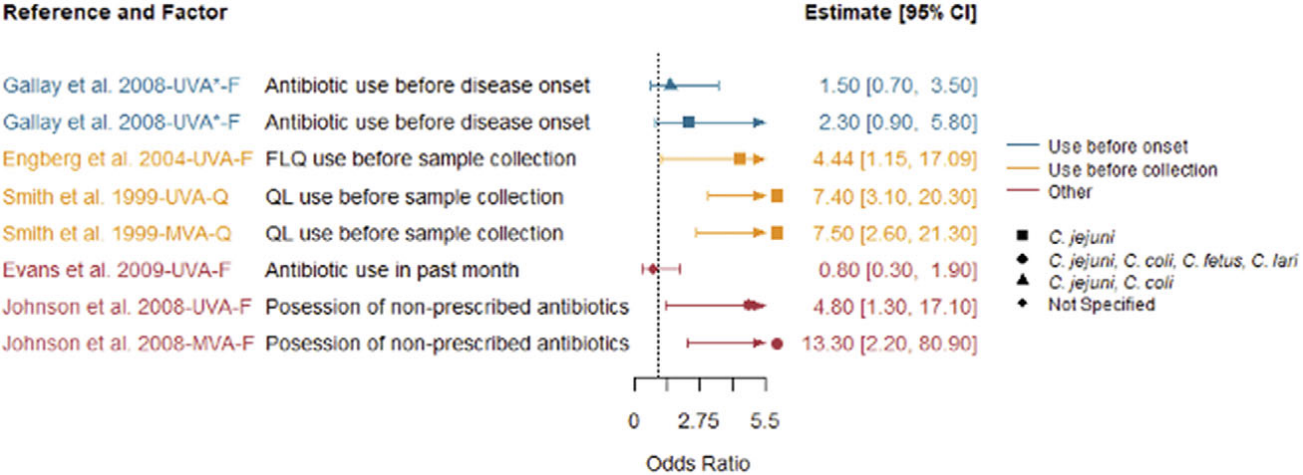
Prior antimicrobial use as factors identified in studies included in the scoping review for human infections with antimicrobial-resistant *Campylobacter* strains compared to infection with susceptible strains, limited to studies reporting odds ratios. *Note*: *F*, fluoroquinolone-resistant outcome; MVA, multivariable analysis result; *Q*, quinolone-resistant outcome; UVA, univariable analysis result; UVA*, results from a study that only conducted univariable analysis.

**Figure 4.**
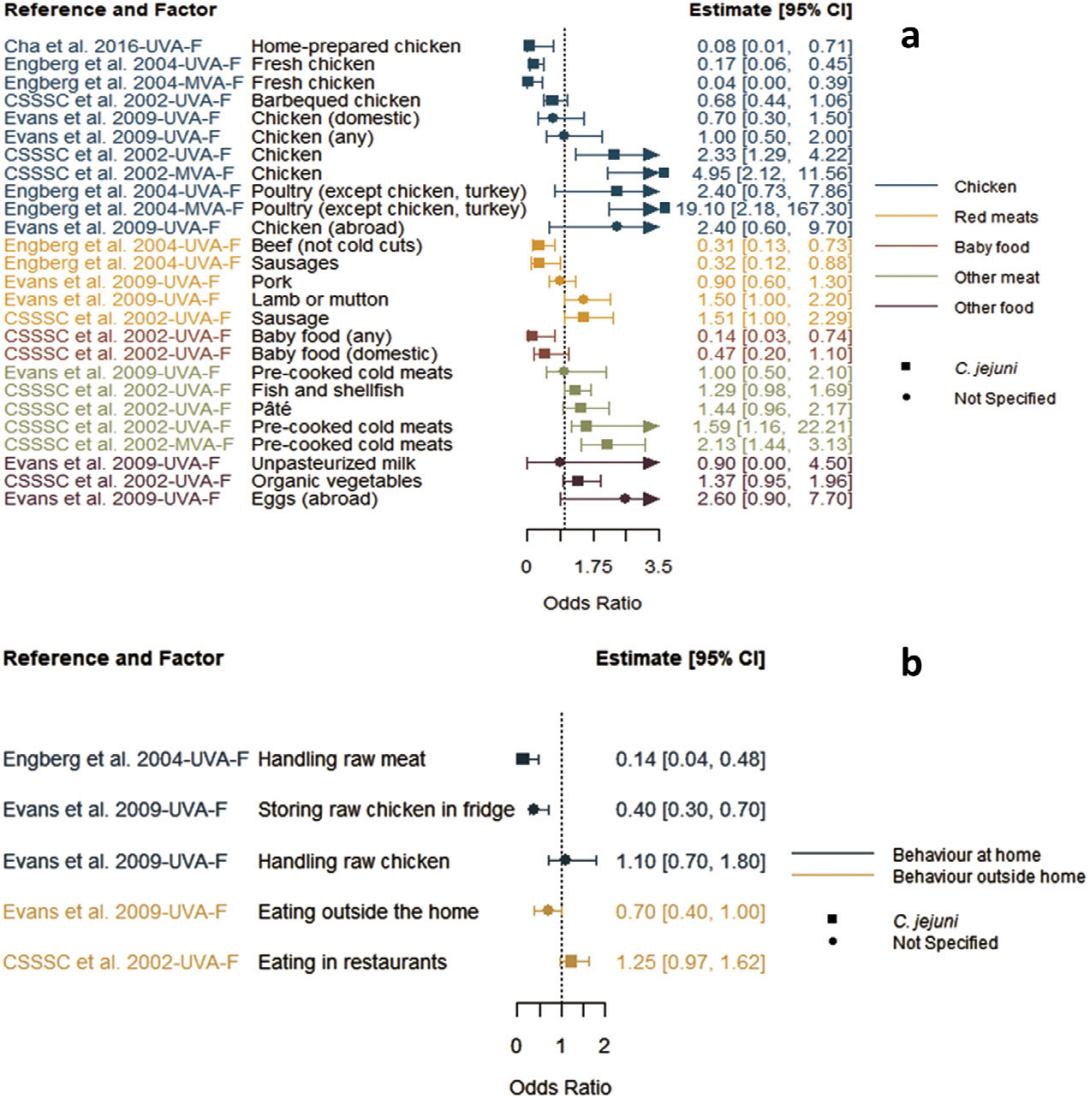
Food consumption (a) and preparation (b) factors identified in studies included in the scoping review for human infections with antimicrobial-resistant *Campylobacter* strains compared to infection with susceptible strains, limited to studies reporting odds ratios. *Note*: CSSSC, *Campylobacter* Sentinel Surveillance Scheme Collaborators; *F*, fluoroquinolone-resistant outcome; MVA, multivariable analysis result; UVA, univariable analysis result.

**Figure 5.**
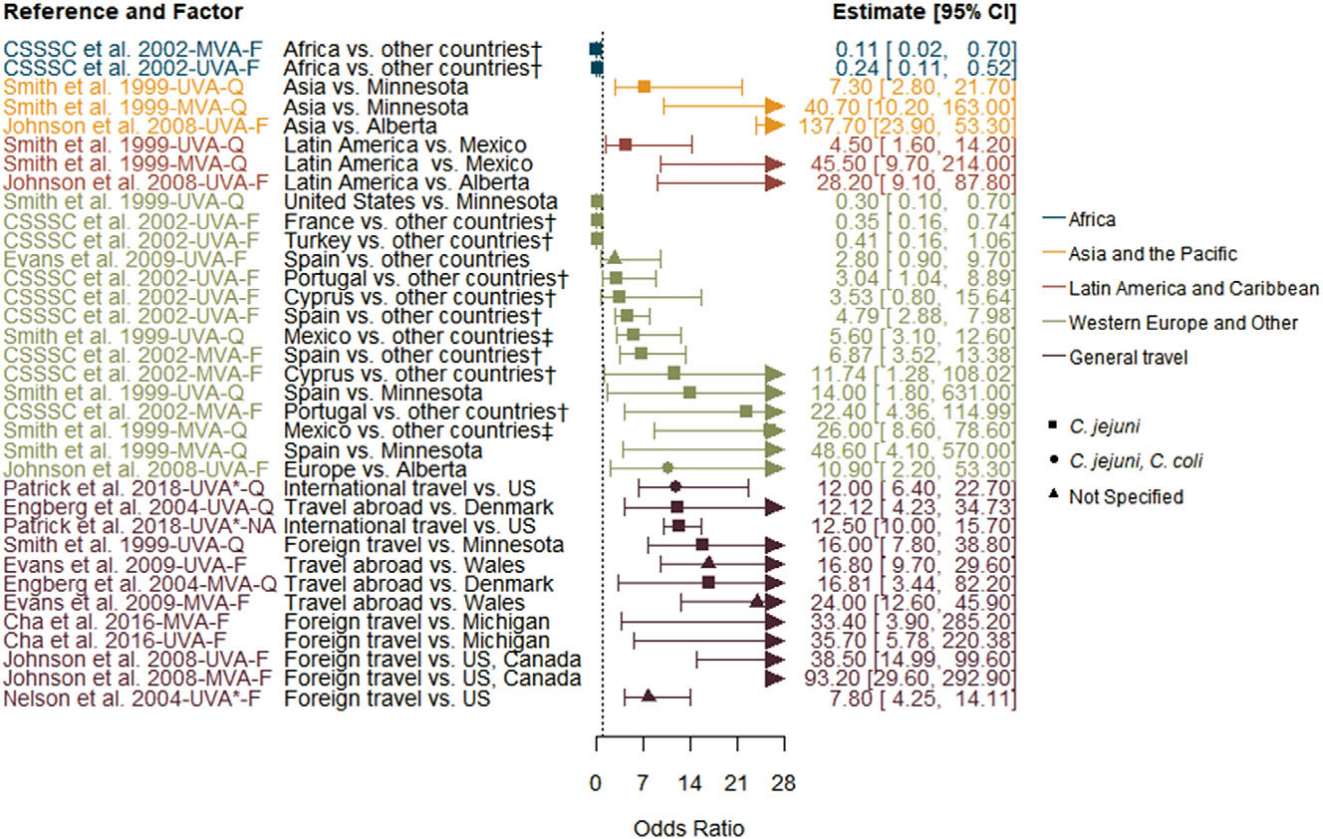
Travel factors identified in studies included in the scoping review for human infections with antimicrobial-resistant *Campylobacter* strains compared to infection with susceptible strains, limited to studies reporting odds ratios. *Note*: CSSSC, *Campylobacter* Sentinel Surveillance Scheme Collaborators; *F*, fluoroquinolone-resistant outcome; MVA, multivariable analysis result; *Q*, quinolone-resistant outcome; UVA, univariable analysis result; UVA*, results from a study that only conducted a univariable analysis.

**Figure 6.**
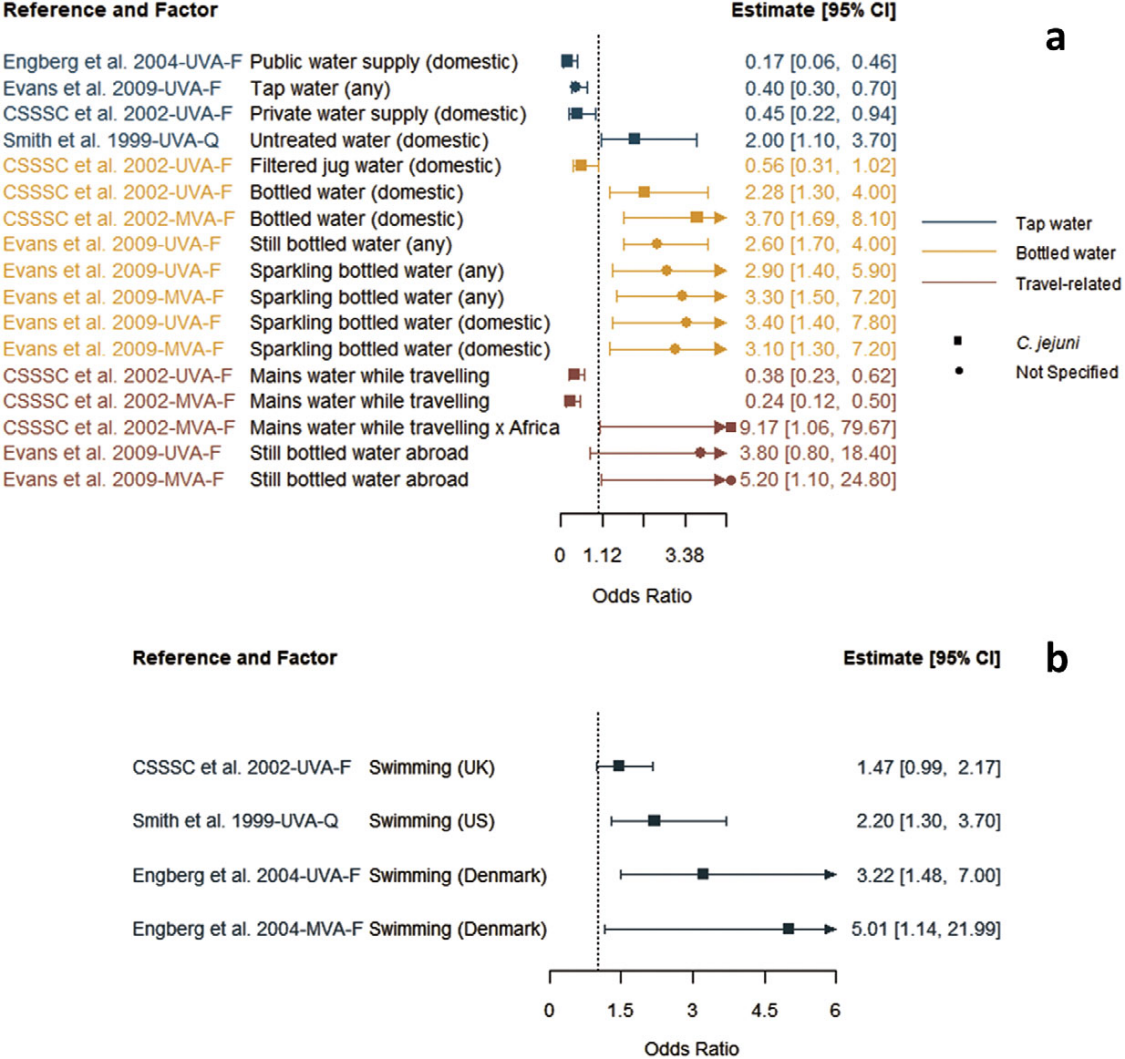
Key data extracted for drinking water-related (a) and swimming (b) factors identified in studies included in the scoping review for human infections with an antimicrobial-resistant *Campylobacter* strains compared to infection with susceptible strains, limited to studies reporting odds ratios. *Note*: CSSSC, *Campylobacter* Sentinel Surveillance Scheme Collaborators; *F*, fluoroquinolone-resistant outcome; MVA, multivariable analysis result; *Q*, quinolone-resistant outcome; UVA, univariable analysis result; UVA*, results from a study that only conducted a univariable analysis.

Reported factors related to resistant *Campylobacter* infections are summarised in Supplementary Table S4 and were combined into seven themes: animal contact (Figure 2), prior antimicrobial use (Figure 3), food and food preparation (Figure 4a,b), travel (Figure 5), underlying health conditions (Supplementary Table S5), water exposure (Figure 6a,b), and participant characteristics (Supplementary Table S5). Articles reporting factors regarding travel (*n* = 17) and participant characteristics (*n* = 14) were the most common. Most of the studies were conducted in a small number of high-income, westernised countries. Studies reported data for unspecified *Campylobacter* species as well as *C. jejuni*, *Campylobacter coli*, *Campylobacter fetus*, and *Campylobacter lari.*

## Synthesis of results

### Animal contact

Five articles reported animal contact as a factor for infection with fluoroquinolone-resistant strains of *Campylobacter* (Figure 2). Most factors, including unspecified pets, pet rodents, dogs, birds, and other domestic or animal contact, were associated with a decreased risk of infection with resistant *Campylobacter* [12, 17–19]. Zoo-animal contact was the only animal factor that was significantly associated with an increased risk [17].

### Prior antimicrobial use

Seven articles reported prior antimicrobial use as a factor [12, 17, 20–24], but only five reported the results of the analysis. All studies with speciated isolates found that prior antimicrobial use was associated with an increased risk of infection with fluoroquinolone-resistant *Campylobacter*, but not all were statistically significant (Figure 3). The study with non-speciated isolates found prior antimicrobial use was associated with a lower risk, but it was not significant. The definition of prior antimicrobial use varied between studies, ranging from possession of non-prescribed antibiotics [21] to the use of an antibiotic before specimen collection [12, 24]. In addition, the definition of the interval for prior antimicrobial use was a month (4 weeks) [12, 17, 20, 24, 25], but when specified, the starting point of this interval also varied from a month prior to the onset of illness [20, 24], the onset of symptoms [12], infection [25], or stool sample collection [21].

### Food and food preparation

Four articles reported many different factors related to food consumption and food handling or associated behaviours, all with fluoroquinolone resistance outcomes (Figure 4a,b) [12, 17–19]. There were opposing results of varying statistical significance for factors such as consumption of chicken, red meat, and other miscellaneous meats, as well as for handling of raw meat and raw chicken at home [12, 17–19] without any discernible patterns. When considering multivariable results, one study reported that those eating chicken had decreased risk, but increased risk when eating poultry other than chicken or turkey [12]. Another reported increased risk when eating chicken or pre-cooked cold meats [18]. Interestingly, two studies found that factors linked to handling [12] or storing of raw chicken [17] were significantly associated with a reduced risk for infection with a fluoroquinolone-resistant strain, whereas the latter paper found no association with handling raw chicken, all from univariable analyses.

### Travel

Seventeen studies reported travel-related factors related to an infection with resistant *Campylobacter* (Figure 5) [11, 12, 17–19, 21, 24–34], and all found foreign travel, regardless of definition and destination country, to be significantly associated with an increased risk of infection with a fluoroquinolone-resistant strain. Of the articles that reported analysis, domestic study populations were limited to the United Kingdom [18], Wales [17], Denmark [12], Canada [21], and the United States [19, 21, 24, 30, 31]. Travel destinations included Africa, Asia, Central and South America, and Europe, but some articles conducted subanalyses on destinations within travel-only cases, which made interpretation challenging [18, 24, 29]. One study considered food and water exposure during travel but did not evaluate travel as a possible interaction [17]. Another study compared the rate of fluoroquinolone-resistant *C. jejuni* infections in Finnish patients that travelled abroad; specifically comparing rates of cases from various travel destinations to those travelling to Thailand [29]. They found that cases in Finnish residents travelling to Spain (including the Canary Islands) and Portugal had lower case rates of fluoroquinolone-resistant infections (rate ratios of 0.11 (95% CI 0.05–0.24) and 0.11 (0.07–0.16), respectively), whereas those travelling to China and India did not differ significantly from Thailand.

### Water

Four articles explored factors related to water exposure, with a focus on water consumption and swimming [12, 17, 18, 24]. There was a large variety in the definition of water consumption-related factors and their association with increased or decreased risk of infection with fluoroquinolone-resistant strains (Figure 6a). Untreated water was associated with increased risk [24], whereas public, tap, or private domestic water was associated with decreased risk [12, 17, 18]. Several bottled water (domestic- or travel-sourced) factors were associated with increased risk [17, 18]. Generally, swimming was reported to increase the risk of infection with a resistant strain (Figure 6b).

### Underlying conditions

Five studies explored factors related to underlying health conditions (Supplementary Table S5), but three did not analyse the data for association [17, 23, 30, 35, 36]. Of the two that did [17, 30], the only statistically significant factor was patients with diabetes (OR 0.30, 95% CI 0.10–1.00) [17]. Antacid use within the past month, indicating other potential conditions, was not significant (OR 1.50, 95% CI 0.90–2.40) [30]. Three studies investigated the risk associated with HIV infection but did not complete the analysis of their data [23, 35, 36].

### Patient characteristics

Thirteen articles explored multiple factors related to participant characteristics such as season of infection, level of education, household income, gender, sex, and age (see Supplementary Table S5) [17–19, 21, 28–31, 37–41]. Factor definitions and results were highly variable, and only four articles conducted multivariable analyses of their data [17–19, 21].

## Discussion

### Summary of evidence

This scoping review identified 27 studies with factors related to human infections with an antimicrobial-resistant strain of *Campylobacter* and provides insight into the available literature and risks associated with these infections. Many reported specific gastrointestinal infections with *C. jejuni*, but there was variability in the site of infection (sample source and *Campylobacter* species), the AMR outcome, and subsequent factor analyses. This review identified key factors associated with infection with resistant strains, such as travel, prior antimicrobial use, animal exposure, and food- and water-related factors, but highlighted the vast heterogeneity of available data and associations with increased or decreased risk of infection with a resistant strain, as well as the gaps that could benefit from further research. Only a small number of studies reported multivariable analysis, and those that did were almost exclusively for fluoroquinolone resistance outcomes. All studies were conducted on cases from a small number of wealthy, westernised countries.

### Risk factors

This review identified several important risk factors associated with human infections with resistant *Campylobacter.* The most consistent was foreign travel, with departure from home countries always being significantly associated with infection with a fluoroquinolone-resistant strain [12, 17, 19, 24, 25, 30, 31, 33, 34]. Care needs to be taken when interpreting these results as only departures from a few wealthy, westernised countries were studied, with highly variable definitions of destinations. Travel is a complex variable that, in this context, is largely a proxy for several different, often unmeasured, factors in the destination country, such as water quality, food/food handling practices and microbial contamination, and potential exposure to different strains of pathogens [42]. Genomics and molecular epidemiology should be employed to better understand the epidemiology of antimicrobial-resistant *Campylobacter* infections in future observational risk-factor studies.

Antimicrobial use prior to infection was another important reported factor for infection with resistant *Campylobacter.* While prior antimicrobial use is recognised to select for AMR, especially in *Campylobacter* [43, 44], only seven of the included studies reported this factor [12, 17, 20–24]. It is possible that many studies did not have access to these data linked to the human cases, which can be difficult to collect/obtain. It is important to note that in these studies, it represents a risk factor for infection with resistant *Campylobacter* compared to susceptible infection, but these observational studies cannot determine whether prior antimicrobial use is specifically selecting for development of a resistant strain in the human host as opposed to selecting for infection with a resistant over a susceptible strain. In addition to the inconsistent definitions of prior antimicrobial use, no studies reported drug dosing or duration, which would be important for future quantitative dose–response modelling. Prior antimicrobial use has been identified as a risk factor for other antimicrobial-resistant, foodborne bacterial infections, such as *Salmonella* Heidelberg [45]. Prior antimicrobial use may be due to inappropriate prescribing or over-the-counter drug access, which may represent less than optimal antimicrobial stewardship [44]. Only one included study reported a factor in this realm, possession of non-prescribed antibiotics [21]. It is also surprising that very few medical conditions requiring antimicrobial use were explored as comorbidities in the included studies. Only three articles looked at HIV and *Campylobacter* and did not analyse their data beyond providing counts [23, 35, 39].

Animal contact, including contact with seemingly healthy pets, has been implicated as a risk factor for AMR in humans [46–48], as well as general human infections with *Campylobacter.* Resistant *Campylobacter* has been isolated from cats and dogs, and pet store puppies have been implicated in a large extensively drug-resistant human outbreak of *C. jejuni* [49, 50]. Conversely, the included studies found that in most cases, animal contact was associated with a reduced risk of infection with a resistant strain compared to susceptible *Campylobacter* [12, 17–19, 24], with the one exception being zoo animal contact [17]. It may be that in these studies, the infecting strains from different animal sources have varying antimicrobial susceptibilities, but these observational study designs were not able to distinguish this, pointing to the need for genomics and molecular epidemiology to better understand these identified associations.

Contaminated foods, especially chicken meat, are known risk factors for infection with *Campylobacter* [4, 51], but only four studies included food-related factors in their analysis [12, 17–19]. There is evidence of AMR spreading to humans through the food chain, specifically broiler chickens in the case of *Campylobacter*, where antimicrobial use on farms may initially select for AMR [16, 52]. However, the results of food-related factors, including chicken, from included studies are mixed and variable. There are several potential reasons for this, including different study populations and potential confounding, intervening, or unmeasured factors, many of which were not considered in studies that did not conduct multivariable analyses. Many studies were cross-sectional, making causal inferences for these relationships challenging. Statistically significant multivariable results for food from two studies were discordant in that one found eating chicken protective while eating other poultry was a risk factor [12]. The other found eating chicken or pre-cooked cold meats to be a risk factor [18]. The food handling results were largely protective, but only from univariable analyses [12, 17]. Some risk factors for infection with *Campylobacter* may be independent of the susceptibility of the strain, and interventions that reduce the overall prevalence or concentration of *Campylobacter* in food or water could also reduce the risk of infection with a resistant strain, yet these types of factors were not studied [53–56]. It is also possible that risk factors for infection with a resistant strain would differ if there was more global representation among the studies included in the review, as different antimicrobials may be used and in some areas, access to these drugs can be over-the-counter for humans and animals [57, 58]. Lastly, while only one study included a factor related to vegetables [18], antimicrobial use in plant agriculture and the use of manure from animals as a fertiliser for crops may increase the risk of resistant organisms on produce [44, 59, 60].

Water consumption and contact are also recognised as potential risk factors for *Campylobacter* infection [51]; however, only four studies reported water-related factors with marked variability in the definition and results [12, 17, 18, 24]. Water contamination with *Campylobacter* varies regionally; however, little is known about contamination with fluoroquinolone-resistant versus susceptible *Campylobacter.* Fluoroquinolone resistance is largely mutational in *Campylobacter*, rather than by acquisition through mobile genetic elements [10], meaning that antimicrobial use in humans or animals and selection for resistant strains that contaminate water is the more likely source compared to acquisition in the environment. None of these studies evaluated this potential linkage, which would require more data about antimicrobial use and genomics.

### Overall considerations

Antimicrobial resistance is a complex, population-level issue across One Health sectors that is driven by individual, regional, and global activities [60]. These studies reported factors at the individual level; however, population-level influences such as environmental sources, cleanliness of water, crop and animal agriculture, the spread of resistant organisms, the overall availability of antimicrobials, and the prescribing nature of the physicians and veterinarians are important to consider [44, 52, 60]. Identified factors and associations with risk of infection with resistant *Campylobacter* were variable, and generalisability was largely limited to wealthy, western countries. Resistance does not recognise borders and AMR surveillance in all countries linked to better patient metadata and genomics are needed to better understand these factors such as travel, prior antimicrobial use, food, and water [42]. In addition to individual patient-level factors, population-level research using a One Health approach that includes water quality, food safety and preparation, and antimicrobial use would expand our knowledge of risk-prevention strategies for infection with resistant *Campylobacter* [60, 61].

Care was taken to state these factors as associations with increased or decreased risk of infection with a resistant strain. The most common study design (cross-sectional) may suffer from reverse causation [62]. Additionally, when evaluating case–control studies, care must be taken when selecting controls to link the factor for AMR and to control for bias [54–56, 61]. The cohort study design controls for the temporality of events and provides the opportunity to measure multiple outcomes, but it is not well suited for the relatively rare incidence of infection with a resistant strain of *Campylobacter* [61].

We chose patients infected with antimicrobial-susceptible strains as our comparison group, which was appropriate for our research question to identify risk factors for infection with a resistant strain among all infections, but may have different results and interpretations than in comparison to healthy patients [61]. This comparison group may not be advantageous for identifying the strength of association for all risk factors of resistant *Campylobacter* infections, especially prior antimicrobial use [56]. Case–case–control studies for infection with resistant organisms compare those infected with a resistant strain to those with a susceptible strain and those who are healthy with a negative test, which allows researchers to better control for bias [54].

Our work yielded less insight into the global understanding of factors associated with human infections with antimicrobial-resistant *Campylobacter* than expected. The dearth of published studies included in our review in any low- and middle-income countries should be a call to action for research funders and government surveillance programmes alike [63]. Tackling AMR requires a One Health approach at the global level [60], and the lack of investment, for example, in AMR surveillance in all but developed countries speaks to the stark gaps present in global AMR research, surveillance, and understanding with a need for an equity lens to be applied to future surveillance and policy. Future use of case–case–control or case–control–control study designs is preferred to examine factors related to infection with resistant strains [54]. Conducting and reporting multivariable analyses is very important as simple univariable associations fail to account for confounding or identify interactions between related factors. In addition, reporting all factors assessed for association, not just those found to be statistically significant in uni- and multivariable models, would provide the complete picture.

### Limitations

We aimed to minimise the possibility of not capturing all eligible articles for our review, a risk inherent in any literature review, by following a rigorous, systematic approach [64]. The factor list identified in this review is by no means exhaustive; it is likely there are factors that were outside the scope of our search or for which research is likely lacking. Our protocol also excluded articles primarily focused on identifying molecular and genetic similarities between human *Campylobacter* isolates with AMR with those from other sources such as animals and water. The synthesis of such literature was beyond the scope of this study but would be an important future contribution to the understanding of human infections with resistant *Campylobacter.* Additionally, excluding non-English articles and publishing bias against null findings has the potential to influence the factors included in our review [14]. There is limited global generalisability because there were no studies from Africa and South America and 24 out of 27 studies were in westernised, high-income countries. The lack of multivariable results for most studies, and, in particular, a seeming lack of identified or assessed interactions between factors, may fail to capture the complicated, interconnected nature of the impact of multiple factors on the risk of infection with resistant strains.

## Conclusions

This scoping review mapped the current literature that investigated and quantified risk or protective factors related to a human infection with antimicrobial-resistant *Campylobacter* compared to susceptible infections. Travel, prior antimicrobial use, food consumption and handling, water consumption and exposure, and animal contact were important factors associated with the risk of infection with a resistant strain. The heterogeneity of the results, focus on fluoroquinolone-resistant outcomes, and lack of multivariable analyses made identifying concrete associations with risk factors challenging but highlighted areas for potential future research. The study of AMR in *Campylobacter* would benefit from an interdisciplinary, One Health research approach that expands to include research in low- and middle-income countries.

## Data Availability

The search protocol and all extracted data are provided in the Supplementary Material. All the Supplementary Material is available on the Cambridge Core website.

## Supplementary material

For supplementary material accompanying this paper visit http://doi.org/10.1017/S0950268823000742. click here to view supplementary material
