# Supplementary material for: A scoping review of factors associated with antimicrobial-resistant Campylobacter species infections in humans
Source: Epidemiol Infect. 2023 Jun 7;151:e100. doi: 10.1017/S0950268823000742 (PMC10311695; doi:10.1017/S0950268823000742)
Supplement: Supplementary file 1 [file S0950268823000742sup001.zip › S0950268823000742sup002.pdf]

Epidemiology and Infection

Supplementary Material

Title: A scoping review of factors associated with antimicrobial-resistant *Campylobacter* species infections in humans

Christine Neustaedter[1,2,3], Dana Tschritter[1,2,3,4], Kelsey Robertson[1,2,3], Richard J. Reid-Smith[3,4,5], Melissa C. MacKinnon[4,5], Colleen P. Murphy[3,4], Carolee A. Carson[3,4], Brennan Chapman[4,5], Norman F. Neumann[1,3], Simon J.G. Otto[1,2,3,6]*

Rest of page intentionally left blank.

Factors associated with antimicrobial resistant *Campylobacter* spp. infections in humans: A scoping review protocol

Authors

Neustaedter C, Reid-Smith RJ, MacKinnon MC, Murphy C, Carson CA, Chapman B, Otto SJG (Final order and author list to be determined)

*Corresponding author: Christine Neustaedter

Author affiliations: University of Alberta: School of Public Health

Email: cneustae@ualberta.ca

Introduction

Campylobacter spp. is one of the leading causes of acute diarrheic illness in the world, accounting for 16% of foodborne illness globally (1) and 8.42% of foodborne illness in Canada (2). Infections are characterized by acute, watery progressing to bloody diarrhea and is often accompanied by abdominal pain, but vomiting is uncommon (3). *Campylobacter* has an incubation period of 2-4 days and most people recover within 2-5 days (4). An uncomplicated infection with *Campylobacter* typically only requires supportive care to avoid dehydration (4). Although uncommon, complications related to *Campylobacter* include but are not limited to bacteremia and increased risk of Guillain-Barré Syndrome (5).

Antimicrobials in the macrolide and quinolone family are commonly used in the treatment of *Campylobacter* infections and have been indicated to reduce duration of illness (6), providing that the infection is susceptible to these antimicrobials. Unfortunately, research indicates that *Campylobacter* has displayed resistance to macrolides, quinolones, and tetracyclines (7). Antimicrobial resistance is commonly defined as the ability of a microorganism to stop an antimicrobial from working against it, rendering standard treatments ineffective (8). Patients with a resistant strain of *Campylobacter* have an increased risk of an adverse health event such as invasive illness or death (9) and may have a higher rate of hospitalization than a susceptible infection (9). Patients with a resistant strain of *Campylobacter* may also experience a longer duration of illness (9).

The purpose of this scoping review is to determine what available published literature exists globally on factors associated with a human infection with antimicrobial resistant *Campylobacter* species. More specifically, factors associated with a human infection with a *Campylobacter* strain that is resistant to the antimicrobials: macrolides; tetracyclines; and/or quinolones, will be investigated. This scoping review will follow the Joanna Briggs Institute Reviewer's Manual framework for scoping reviews which defines the objectives, methods and reporting of the review to facilitate a transparent review process (10).

The term antimicrobial resistance (AMR) used in this review will refer to the ability of microorganisms, such as bacteria, fungi, or viruses, to withstand, to varying extents, the effects of an antimicrobial to which they were formerly susceptible (11). The search will not be limited by specific *Campylobacter* species, and resistance must be determined by recognized laboratory antimicrobial susceptibility testing methods. Factors will be assessed during the screening process using the intentionally broad definition of a practice or circumstance that positively or negatively influences the occurrence of AMR (12). This definition does not consider the concept of causality; we consider any relationship between an exposure and outcome as a factor, whether or not a causal pathway is present (12).

A preliminary search for existing reviews and relevant research was completed on January 21, 2020 in Ovid Medline®, Cochrane Library, Joanna Briggs Institute Systematic Review Registry, and Google Scholar. No scoping or systematic reviews were found but there were indications that relevant research exists. The objective of this review will be to gather existing research on risk factors for a human infected with an antimicrobial resistant *Campylobacter* species and characterize the body of evidence thereby establishing the array of risk factors in addition to exploring the variability between the studies.

Inclusion Criteria

Types of Participants

Any study that evaluates humans of any age with a *Campylobacter* infection (confirmed by recognized laboratory methods) will be included. Non-human research, studies that evaluate infections other than *Campylobacter*, studies that evaluate colonization instead of infection, and studies that fail to confirm a *Campylobacter* infection by culture will be excluded.

Concept

Studies must evaluate the exposure of interest which are factors involved with a human infection with a resistant *Campylobacter* strain, for example, known risk factors of age, recent travel, pre-existing medical conditions, but the search won't be limited to those mentioned. Studies which do not evaluate factors related to a human infection will be excluded. The comparator group will be appropriate to the study design. For example, when applicable, the comparator group for case-control studies will be infections with *Campylobacter* that are susceptible to the antimicrobials of interest. Our outcome of interest are studies evaluating resistance to our antimicrobials of interest: macrolides, tetracyclines, and quinolones. Resistance must be determined by recognized laboratory antimicrobial susceptibility testing methods.

Context

There were no limits applied to language, geographical location, *Campylobacter* species, and date published. Non-English articles will be identified and excluded during primary screening.

Types of Studies

The review will include any analytic study, including theses and dissertations. Study designs that will be excluded from the review are: review articles, commentary, opinion pieces, editorials, newspaper articles, books, book chapters, and conference proceedings.

Search Strategy

An initial limited search was completed in MEDLINE® in Ovid using a preliminary search string (13-15). An informal analysis of terms used in the title, abstract and index was conducted and relevant terms were included in the search string. The final search string, see appendix, will be adapted and applied across all of the following databases: AGRICOLA™ in ProQuest®, Centre for Agriculture and Bioscience abstracts in Web of Science, EMBASE® in Ovid, Scopus®, and MEDLINE® in Ovid. Grey literature sources to be searched will be the World Health Organization's Global Index Medicus, and the Bielefield Academic Search Engine. Additionally, the first 250 results, sorted based on relevance, from Google Scholar will also be screened for eligibility. Lastly, the lists of references from the included studies will be reviewed to identify any additional relevant articles.

Study Selection

The primary screening will be conducted on the title and abstract of each article. The following questions will be used in the primary screening process:

1. Does the title and/or abstract indicate the study subjects are human?
2. Does the title and/or abstract indicate the study participants have *Campylobacter* infections?
3. Does the title and/or abstract indicate at least some of the study participants have a *Campylobacter* infection that is resistant to the antimicrobials of interest?
4. Does the title and/or abstract indicate that the study reports on the effect of a factor on resistance?
5. Is the study published in English?

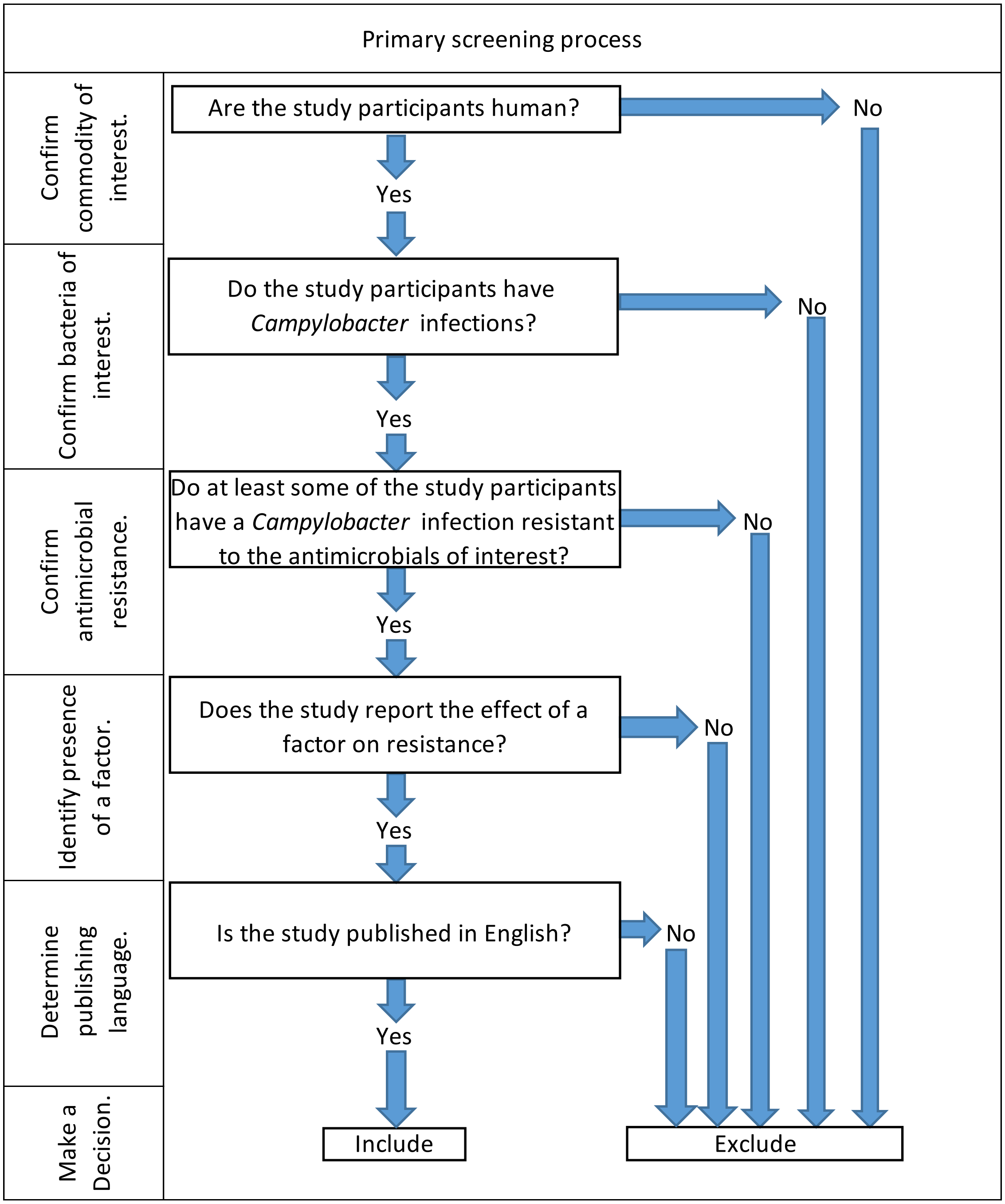

The primary screening will be performed by two researchers. The possible answers are yes, no, an unclear. If the article fully or partially meets the inclusion criteria (i.e. all screening questions are answered either 'yes' or 'unclear'), the article will proceed to secondary screening. Reasons for exclusion will be indicated. For both primary and secondary screening, the answers will be compared and any disagreements will be discussed until consensus is achieved. If consensus cannot be reached, then a third researcher will be used to arbitrate.

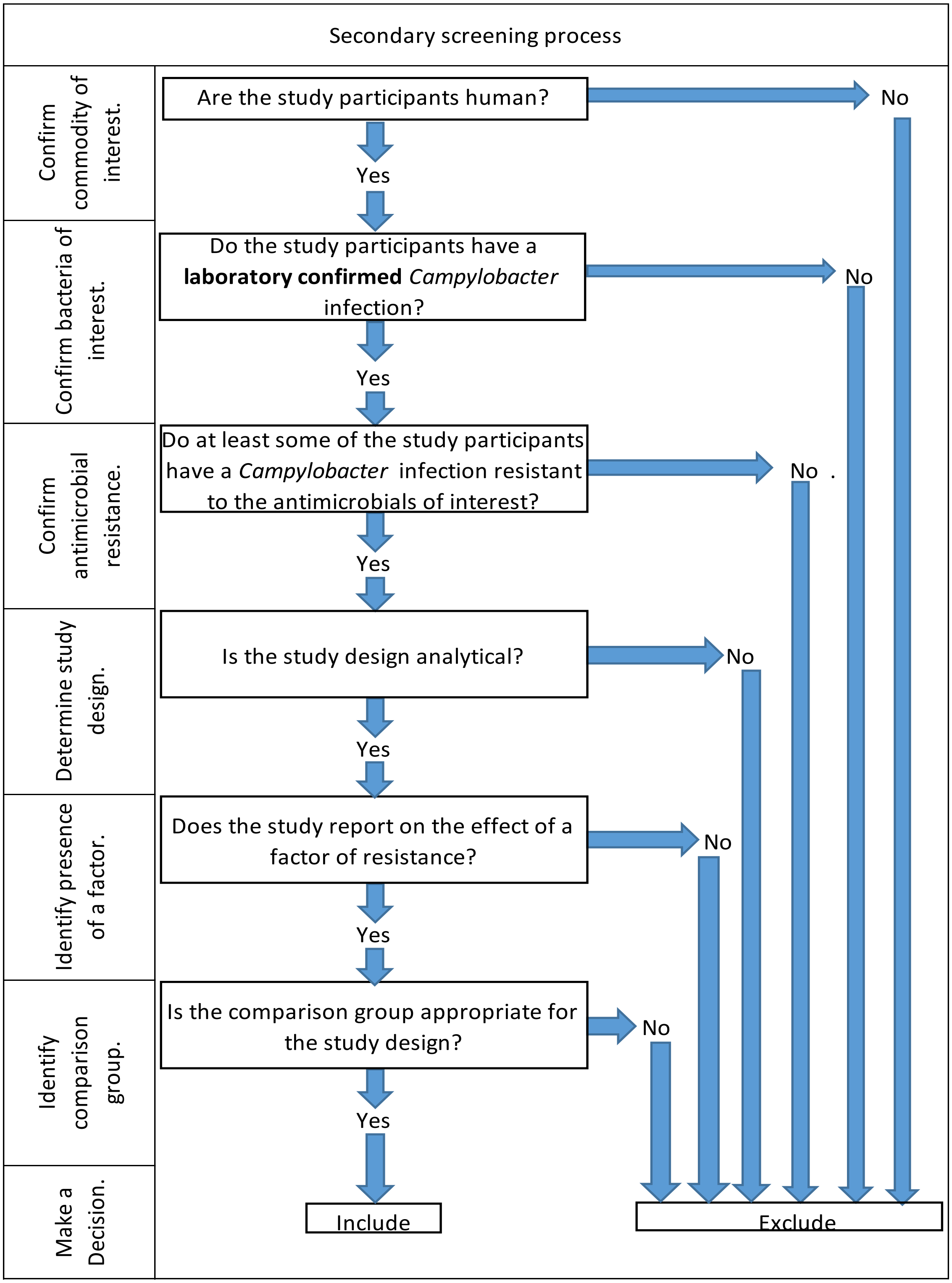

The secondary screening will be performed on the full text articles. The following questions will be used for the secondary screening:

1. Are study subjects are human?
2. Do the study participants have a laboratory confirmed *Campylobacter* infection?

3. Do at least some of the study participants have a *Campylobacter* infection that is resistant to the antimicrobials of interest?
4. Is the study design analytical?
5. Does the study report on the effect of a factor on resistance?
6. Is the comparison group appropriate for the study design?

The secondary screening will be performed independently by two researchers. The possible answers are 'yes' or 'no'. For an article to be included, all of the above questions must be answered with a 'yes'. One or more answers of 'no' to the questions above leads to exclusion of the article and information regarding the reason for exclusion will be recorded.

Data Extraction

EndNote X9™ will be used for managing citations. All eligible articles will be uploaded onto EndNote X9™ and any duplicates will be removed. The remaining articles will then be uploaded onto DistillerSR® and checked again for duplicates. Screening forms will be created using the software and will be used to determine eligibility.

A data extraction form will be created in DistillerSR®. Data extracted will include:

- Characteristics of the study, including:
    - Year of publication
    - Type of document
    - Author report study design
    - Year(s) data were collected
    - Country or countries study was performed in
    - Methods used
- Characteristics of the study participants, including:
    - Population
    - Sample size
- Description of and results for factor(s) investigated, including:
    - Count and rate data
    - Measures of association and variation
    - Definition of factor(s) investigated
    - Associated results and key findings

Presentation of Results

Results will be presented using a narrative summary with the inclusion of tables and summaries of results as is warranted. Narrative summaries will be completed for each type of resistance of interest. Tables will include characteristics of the studies, characteristics of the participants, and the factors investigated.

References

(1) World Health Organization. WHO Estimates of the Global Burden of Foodborne Diseases. 2015 (cited November 29. 2019): 71. Available from: https://www.who.int/foodsafety/publications/foodborne_disease/fergreport/en/

(2) Thomas MK, Murray R, Flockhart L, Pintar K, Pollari F, Fazil A, Nesbitt A, Marshall B. Estimates of the burden of foodborne illness in Canada for 30 specified pathogens and unspecified agents, circa 2006. Foodborne Pathog Dis; 10(7):639-48.

(3) Moore JE, Corcoran D, Dooley JSG, Fanning S, Lucey B, Matsuda M, et al. Campylobacter. Vet Res 2005;36(3):351-382.

(4) Centers for Disease Control and Prevention. *Campylobacter* (Campylobacteriosis). 2017 (cited December 9, 2019). Available from: https://www.cdc.gov/campylobacter/technical.html

(5) Nachamkin I, Mishu Allos B, Ho T. *Campylobacter* species and Guillain-Barré syndrome. Clin Microbiol Rev 1998; 11(3): 555-567.

(6) Ternhag A, Asikainen T, Giesecke J, Ekdahl K. A meta-analysis on the effects of antibiotic treatment on duration of symptoms caused by infection with Campylobacter species. Clin Infect Dis 2007; 44(5): 696-700.

(7) Luangtongkum T, Byeonghwa J, Han J, Plummer P, Logue CM, Zhang Q. Antibiotic resistance in *Campylobacter*: Emergence, transmission and persistence. Future Microbiol 2009; 4(2): 189-200.

(8) World Health Organization. Antimicrobial resistance. 2019 (cited 2019 November 29). Available from: https://www.who.int/antimicrobial-resistance/en/

(9) Helms M, Simonsen J, Olsen KEP, Mølbak K. Adverse health events associated with antimicrobial drug resistance in Campylobacter species: a registry-based cohort study. J Infect Dis 2005; 191(7): 1050-5.

(10) Peters MDJ, Godfrey C, McInerney P, Baldini Soares C, Khalil H, Parker D. Chapter 11: Scoping Reviews. In: Aromataris E, Munn Z (Editors). *Joanna Briggs Institute reviewer's manual.* The Joanna Briggs Institute, 2017. Available from https://reviewersmanual.joannabriggs.org/

(11) World Health Organization. Antimicrobial resistance. 2019 (cited 2019 July 16). Available from: https://www.who.int/antimicrobial-resistance/en/

(12) The IAM.AMR Project Documentation. Literature search. 2019. Available from: http://docs.grdi-amr.com/en/latest/project/search.html

(13) MacKinnon M, Sargeant J, Pearl D, Reid-Smith R, Carson C, Parmley J, McEwen S. A protocol for a systematic review and meta-analysis of the health and healthcare system burden due to human *Escherichia coli* infections resistant to third/fourth/fifth generation cephalosporins or quinolones, or with multidrug resistance. PROSPERO, 2018 CRD42018111197. Available from: http://www.crd.york.ac.uk/PROSPERO/display_record.php?ID=CRD42018111197

(14) De Jongh EJ, Harper SL, Yamamoto S, Wright CJ, Otto SJG. One world, one hive: A scoping review of honey bees, climate change, pollutants, and antimicrobial resistance. University of Alberta, Edmonton; 2019 (Unpublished).

(15) Murphy C., Mercucci K, Chapman, B., Carson, C. AMR in livestock for meat consumption. Federal Health Library, Canada; 2019 (Unpublished).

(16) Murphy CP, Carson C, Marleau J, Reid-Smith R, Chapman B. Comparative human exposure to antimicrobial-resistant *Campylobacter*, *Escherichia coli*, *Salmonella enterica* & nbsp from food animals using integrated assessment modelling: A farm to fork approach. The 5th International One Health Congress, 2018 June 23,(Conference Presentation):1.

APPROVED

By Christine Neustaedter at 5:56 pm, Feb 04, 2020

Appendix

Ovid MEDLINE®	
#	Searches
1	Campylobacter$.ab,kf,ti. or exp Campylobacter/
2	exp Drug Resistance, Microbial/ or resistan$.ab,kf,ti.
3	(Chlortetracycline$ or clomocycline$ or demeclocycline$ or doxycycline$ or eravacycline$ or lymecycline$ or metacycline$ or minocycline$ or omadacycline$ or oxytetracycline$ or penimepicycline$ or rolitetracycline$ or tetracycline$).ab,kf,ti.
4	(azithromycin$ or cethromycin$ or clarithromycin$ or dirithromycin$ or erythromycin$ or fidaxomicin$ or flurithromycin$ or gamithromycin$ or josamycin$ or kitasamycin$ or midecamycin$ or miocamycin$ or oleandomycin$ or rokitamycin$ or roxithromycin$ or spiramycin$ or telithromycin$ or tildipirosin$ or tilmicosin$ or troleandomycin$ or solithromycin$ or tulathromycin$ or tylosin$ or tylvalosin$ or macrolide$).ab,kf,ti.
5	(besifloxacin$ or cinoxacin$ or ciprofloxacin$ or danofloxacin$ or delafloxacin$ or difloxacin$ or enoxacin$ or enrofloxacin$ or fleroxacin$ or flumequine$ or garenoxacin$ or gatifloxacin$ or gemifloxacin$ or grepafloxacin$ or ibafloxacin$ or levofloxacin$ or lomefloxacin$ or marbofloxacin$ or moxifloxacin$ or nadifloxacin$ or nalidixic acid or norfloxacin$ or ofloxacin$ or orbifloxacin$ or ozenoxacin$ or oxolinic acid or pazufloxacin$ or pefloxacin$ or pipemidic acid or piromidic acid or pradofloxacin$ or prulifloxacin$ or rosoxacin$ or rufloxacin$ or sitafloxacin$ or sparfloxacin$ or temafloxacin$ or quinolone$ or fluoroquinolone$).ab,kf,ti.
6	Drug Resistance, Multiple, Bacterial/ or (MDR or XDR or PDR or important antimicrobial$ or important antibiotic$).ab,kf,ti.
7	2 or 3 or 4 or 5 or 6
8	1 and 7
9	8 not (Animal/ not (Animal/ and Human/))

Note: Search string development used references 13-15

ProQuest ® AGRICOLA™	
#	Searches
1	noft(Campylobacter*)
2	noft(“Drug Resistance” or resistan*)
3	noft(Chlortetracycline* or clomocycline* or demeclocycline* or doxycycline* or eravacycline* or lymecycline* or metacycline* or minocycline* or omadacycline* or oxytetracycline* or penimepicycline* or rolitetracycline* or tetracycline*)
4	noft(azithromycin* or cethromycin* or clarithromycin* or dirithromycin* or erythromycin* or fidaxomicin* or flurithromycin* or gamithromycin* or josamycin* or kitasamycin* or midecamycin* or miocamycin* or oleandomycin* or rokitamycin* or roxithromycin* or spiramycin* or telithromycin* or tildipirosin* or tilmicosin* or troleandomycin* or solithromycin* or tulathromycin* or tylosin* or tylvalosin* or macrolide*)
5	noft(besifloxacin* or cinoxacin* or ciprofloxacin* or danofloxacin* or delafloxacin* or difloxacin* or enoxacin* or enrofloxacin* or fleroxacin* or

	flumequine* or garenoxacin* or gatifloxacin* or gemifloxacin* or grepafloxacin* or ibafloxacin* or levofloxacin* or lomefloxacin* or marbofloxacin* or moxifloxacin* or nadifloxacin* or nalidixic acid or norfloxacin* or ofloxacin* or orbifloxacin* or ozenoxacin* or oxolinic acid or pazufloxacin* or pefloxacin* or pipemidic acid or piromidic acid or pradofloxacin* or prulifloxacin* or rosoxacin* or rufloxacin* or sitafloxacin* or sparfloxacin* or temafloxacin* or quinolone* or fluoroquinolone*)
6	noft(MDR or XDR or PDR or “important antimicrobial*” or “important antibiotic*”)
7	S2 or S3 or S4 or S5 or S6
8	S1 and S7
9	S8 NOT (noft(nonhuman* or animal*) NOT noft(((human*) AND ((nonhuman*) OR (“animal*”)))))

Web of Science: CABI: CAB Abstracts® and Global Health®	
#	Searches
1	TS=(Campylobacter*) or DE=(Campylobacter*)
2	TS=(“Drug Resistance” or resistan*) or DE=(“drug resistance”)
3	TS=(Chlortetracycline* or clomocycline* or demeclocycline* or doxycycline* or eravacycline* or lymecycline* or metacycline* or minocycline* or omadacycline* or oxytetracycline* or penimepicycline* or rolitetracycline* or tetracycline*)
4	TS=(azithromycin* or cethromycin* or clarithromycin* or dirithromycin* or erythromycin* or fidaxomicin* or flurithromycin* or gamithromycin* or josamycin* or kitasamycin* or midecamycin* or miocamycin* or oleandomycin* or rokitamycin* or roxithromycin* or spiramycin* or telithromycin* or tildipirosin* or tilmicosin* or troleandomycin* or solithromycin* or tulathromycin* or tylosin* or tylvalosin* or macrolide*)
5	TS=(besifloxacin* or cinoxacin* or ciprofloxacin* or danofloxacin* or delafloxacin* or difloxacin* or enoxacin* or enrofloxacin* or fleroxacin* or flumequine* or garenoxacin* or gatifloxacin* or gemifloxacin* or grepafloxacin* or ibafloxacin* or levofloxacin* or lomefloxacin* or marbofloxacin* or moxifloxacin* or nadifloxacin* or nalidixic acid or norfloxacin* or ofloxacin* or orbifloxacin* or ozenoxacin* or oxolinic acid or pazufloxacin* or pefloxacin* or pipemidic acid or piromidic acid or pradofloxacin* or prulifloxacin* or rosoxacin* or rufloxacin* or sitafloxacin* or sparfloxacin* or temafloxacin* or quinolone* or fluoroquinolone*)
6	TS=(MDR or XDR or PDR or “important antimicrobial*” or “important antibiotic*”) or DE=(“multiple drug resistance”)
7	#2 or #3 or #4 or #5 or #6
8	#1 and #7
9	((TS=animal* NOT (TS=(animal* AND human*))))
10	#8 NOT #9

Ovid EMBASE®	
#	Searches
1	campylobacter$.ab,kw,ti. or exp campylobacter/
2	exp antibiotic resistance/ or resistan$.ab,kw,ti.
3	(chlortetracycline$ or clomocycline$ or demeclocycline$ or doxycycline$ or eravacycline$ or lymecycline$ or metacycline$ or minocycline$ or

	omadacycline$ or oxytetracycline$ or penimepicycline$ or rolitetracycline$ or tetracycline$).ab,kw,ti.
4	(azithromycin$ or cethromycin$ or clarithromycin$ or dirithromycin$ or erythromycin$ or fidaxomicin$ or flurithromycin$ or gamithromycin$ or josamycin$ or kitasamycin$ or midecamycin$ or miocamycin$ or oleandomycin$ or rokitamycin$ or roxithromycin$ or spiramycin$ or telithromycin$ or tildipirosin$ or tilmicosin$ or troleandomycin$ or solithromycin$ or tulathromycin$ or tylosin$ or tylvalosin$ or macrolide$).ab,kw,ti.
5	(besifloxacin$ or cinoxacin$ or ciprofloxacin$ or danofloxacin$ or delafloxacin$ or difloxacin$ or enoxacin$ or enrofloxacin$ or fleroxacin$ or flumequine$ or garenoxacin$ or gatifloxacin$ or gemifloxacin$ or grepafloxacin$ or ibafloxacin$ or levofloxacin$ or lomefloxacin$ or marbofloxacin$ or moxifloxacin$ or nadifloxacin$ or nalidixic acid or norfloxacin$ or ofloxacin$ or orbifloxacin$ or ozenoxacin$ or oxolinic acid or pazufloxacin$ or pefloxacin$ or pipemidic acid or piromidic acid or pradofloxacin$ or prulifloxacin$ or rosoxacin$ or rufloxacin$ or sitafloxacin$ or sparfloxacin$ or temafloxacin$ or quinolone$ or fluoroquinolone$).ab,kw,ti.
6	exp multidrug resistance/ or (MDR or XDR or PDR or important antimicrobial$ or important antibiotic$).ab,kw,ti.
7	2 or 3 or 4 or 5 or 6
8	1 and 7
9	8 NOT ((exp animal/ or nonhuman/) not exp human/)

Scopus®	
#	Searches
1	TITLE-ABS-KEY(Campylobacter*)
2	TITLE-ABS-KEY(“Drug Resistance” or resistan*)
3	TITLE-ABS-KEY(Chlortetracycline* or clomocycline* or demeclocycline* or doxycycline* or eravacycline* or lymecycline* or metacycline* or minocycline* or omadacycline* or oxytetracycline* or penimepicycline* or rolitetracycline* or tetracycline*)
4	TITLE-ABS-KEY(azithromycin* or cethromycin* or clarithromycin* or dirithromycin* or erythromycin* or fidaxomicin* or flurithromycin* or gamithromycin* or josamycin* or kitasamycin* or midecamycin* or miocamycin* or oleandomycin* or rokitamycin* or roxithromycin* or spiramycin* or telithromycin* or tildipirosin* or tilmicosin* or troleandomycin* or solithromycin* or tulathromycin* or tylosin* or tylvalosin* or macrolide*)
5	TITLE-ABS-KEY(besifloxacin* or cinoxacin* or ciprofloxacin* or danofloxacin* or delafloxacin* or difloxacin* or enoxacin* or enrofloxacin* or fleroxacin* or flumequine* or garenoxacin* or gatifloxacin* or gemifloxacin* or grepafloxacin* or ibafloxacin* or levofloxacin* or lomefloxacin* or marbofloxacin* or moxifloxacin* or nadifloxacin* or “nalidixic acid” or norfloxacin* or ofloxacin* or orbifloxacin* or ozenoxacin* or “oxolinic acid” or pazufloxacin* or pefloxacin* or “pipemidic acid” or “piromidic acid” or pradofloxacin* or prulifloxacin* or rosoxacin* or rufloxacin* or sitafloxacin* or sparfloxacin* or temafloxacin* or quinolone* or fluoroquinolone*)
6	TITLE-ABS-KEY(MDR or XDR or PDR or “important antimicrobial*” or “important antibiotic*”)
7	#2 or #3 or #4 or #5 or #6

8	#1 and #7
9	TITLE-ABS-KEY(animal*) AND NOT (TITLE-ABS-KEY(animal* AND human*))
10	#8 AND NOT #9

Grey Literature	
Search Engine	Search String
Google Scholar	campylobacter* AND (resistance or fluoroquinolone or macrolide or tetracycline)
Global Index Medicus (WHO)	campylobacter* AND (resistance or fluoroquinolone or tetracycline or macrolide)
Bielefield Academic Search Engine	((campylobacter*) AND (resistance fluoroquinolone macrolide tetracycline)) NOT (animal* NOT (human* AND animal*))
