# Supplementary material for: A scoping review of factors associated with antimicrobial-resistant Campylobacter species infections in humans
Source: Epidemiol Infect. 2023 Jun 7;151:e100. doi: 10.1017/S0950268823000742 (PMC10311695; doi:10.1017/S0950268823000742)
Supplement: Supplementary file 1 [file S0950268823000742sup001.zip › S0950268823000742sup003.pdf]

Table S2. Search string used to search the MEDLINE® via Ovid® database for articles about factors associated with human infection with an antimicrobial-resistant strain of *Campylobacter* species.

Component	Searches
**(1) Organism**	Campylobacter$.ab,kf,ti. or exp Campylobacter/
**(2) Antimicrobial resistance**	exp Drug Resistance, Microbial/ or resistan$.ab,kf,ti.
**(3) Antimicrobials**	(Chlortetracycline$ or clomocycline$ or demeclocycline$ or doxycycline$ or eravacycline$ or lymecycline$ or metacycline$ or minocycline$ or omadacycline$ or oxytetracycline$ or penimepicycline$ or rolitetracycline$ or tetracycline$).ab,kf,ti.
**(4) Antimicrobials**	(azithromycin$ or cethromycin$ or clarithromycin$ or dirithromycin$ or erythromycin$ or fidaxomicin$ or flurithromycin$ or gamithromycin$ or josamycin$ or kitasamycin$ or midecamycin$ or miocamycin$ or oleandomycin$ or rokitamycin$ or roxithromycin$ or spiramycin$ or telithromycin$ or tildipirosin$ or tilmicosin$ or troleandomycin$ or solithromycin$ or tulathromycin$ or

**(5) Antimicrobials**	(besifloxacin$ or cinoxacin$ or ciprofloxacin$ or danofloxacin$ or delafloxacin$ or difloxacin$ or enoxacin$ or enrofloxacin$ or fleroxacin$ or flumequine$ or garenoxacin$ or gatifloxacin$ or gemifloxacin$ or grepafloxacin$ or ibafloxacin$ or levofloxacin$ or lomefloxacin$ or marbofloxacin$ or moxifloxacin$ or nadifloxacin$ or nalidixic acid or norfloxacin$ or ofloxacin$ or orbifloxacin$ or ozenoxacin$ or oxolinic acid or pazufloxacin$ or pefloxacin$ or pipemidic acid or piromidic acid or pradofloxacin$ or prulifloxacin$ or rosoxacin$ or rufloxacin$ or sitafloxacin$ or sparfloxacin$ or
**(6) Antimicrobial Resistance**	Drug Resistance, Multiple, Bacterial/ or (MDR or XDR or PDR or important antimicrobial$ or important antibiotic$).ab,kf,ti.
**(7)**	2 or 3 or 4 or 5 or 6
**(8)**	1 and 7
**(9)**	8 not (Animal/ not (Animal/ and Human/))
