# Supplementary material for: A scoping review of factors associated with antimicrobial-resistant Campylobacter species infections in humans
Source: Epidemiol Infect. 2023 Jun 7;151:e100. doi: 10.1017/S0950268823000742 (PMC10311695; doi:10.1017/S0950268823000742)
Supplement: Supplementary file 1 [file S0950268823000742sup001.zip › S0950268823000742sup004.pdf]

Table S4. Factors regarding human infection with an antimicrobial-resistant strain of *Campylobacter* species reported in studies included in the scoping review*.

Category	Factor	# of relevant articles	Reference
Animal contact		5	
	Pets	4	[17-19, 24]
	Zoo animals	1	[17]
	Unspecified	1	[12]
Prior Antimicrobial Use		7	[12, 17, 20-22, 24, 25]
Patient/infection characteristics		13	
	Age	8	[17-19, 21, 28, 31, 39, 41]
	Gender/Sex	6	[17, 19, 21, 28, 30, 40]
	Education	3	[17, 21, 30]
	Geography	3	[21, 30, 31]
	Season of infection	3	[18, 19, 21]
	Employment status	2	[17, 18]
	Men who have sex with men	2	[37, 38]
	Race	2	[30, 31]
	Species of *Campylobacter*	2	[21, 31]
	Contact with children	1	[17]
	Income	1	[30]
	Year of collection	1	[29]
Food and Food Preparation		4	
	Chicken	4	[12, 17-19]

	Cold cuts	2	[17, 18]
	Eating away from home	2	[17, 18]
	Sausages	2	[12, 18]
	Baby food	1	[18]
	Barbequed	1	[18]
	Beef	1	[12]
	Eggs	1	[17]
	Fish and shellfish	1	[18]
	Handling of raw chicken	1	[17]
	Handling of raw meat	1	[12]
	Lamb or mutton	1	[17]
	Organic Vegetables	1	[18]
	Pâté	1	[18]
	Pork	1	[17]
	Poultry (excl. chicken or turkey)	1	[12]
	Storing raw chicken in fridge	1	[17]
	Unpasteurized milk	1	[17]
Travel		16	
	Travel (general)	13	[11, 12, 17-19, 21, 24, 25, 27, 30-33]
	Asia	6	[24, 26, 28, 29, 34]
	Europe	5	[17, 18, 24, 29, 34]
	Africa	4	[18, 26, 28, 34]

	Latin America	2	[18, 26]
	North America	2	[18, 24]
	Arabian Peninsula	1	[28]
	Australia/New Zealand	1	[18]
	Ill contact who traveled	1	[17]
	North Africa-Middle East	1	[26]
Underlying Conditions		5	
	HIV	3	[23, 35, 36]
	Antacid use	1	[17]
	Diabetic	1	[17]
	Unspecified	1	[30]
Water		4	
	Swimming	3	[12, 18, 24]
	Bottled water	2	[17, 18]
	Public water supply	2	[12, 18]
	Filtered jug water	1	[18]
	Private water supply	1	[18]
	Sparkling bottled water	1	[17]
	Tap water	1	[17]
	Untreated water	1	[24]

* This table includes factors that were investigated, regardless of interpretation of statistical significance. References may report more than one factor, and as a result the number of relevant articles for each category may not sum.
