# Supplementary material for: A scoping review of factors associated with antimicrobial-resistant Campylobacter species infections in humans
Source: Epidemiol Infect. 2023 Jun 7;151:e100. doi: 10.1017/S0950268823000742 (PMC10311695; doi:10.1017/S0950268823000742)
Supplement: Supplementary file 1 [file S0950268823000742sup001.zip › S0950268823000742sup005.pdf]

Table S5. Patient characteristic factors* identified in studies included in the scoping review for human infection with antimicrobial-resistant *Campylobacter* compared to infection with susceptible strains.

Category	Factor	Species	AMR outcome	Univariable Result	Multivariable Result	Reference
**Social determinants of health**						
	Residence: urban/suburban vs other	Not specified	Fluoroquinolones	p=0.02	No analysis	Nelson J, et al, 2004
	Rural residence	*jejuni, coli,* other	Fluoroquinolones	p=0.02	No analysis	Johnson J, et al, 2008
	Metro vs. suburban and rural areas	*jejuni*	Quinolones<br>Macrolides	No analysis	No analysis	Patrick M, et al, 2018
	Health region	*jejuni, coli,* other	Fluoroquinolones	p=0.01	No analysis	Johnson J, et al, 2008
	Education: bachelor's degree or higher vs other	Not specified	Fluoroquinolones	$p<0.01$	No analysis	Nelson J, et al, 2004
	College or university education	*jejuni, coli,* other	Fluoroquinolones	p=0.40	No analysis	Johnson J, et al, 2008
	Household income: >60,000 vs lower	Not specified	Fluoroquinolones	p=0.02	No analysis	Nelson J, et al, 2004
	Employment status 1: employed	Not specified	Fluoroquinolones	OR = 1.8 (1.2-2.6), p=0.01	No analysis [a]	Evans M, et al, 2009
	Employment status 2: student	Not specified	Fluoroquinolones	OR = 0.2 (0.0-0.7), p=0.02	No analysis [a]	Evans M, et al, 2009

Category	Factor	Species	AMR outcome	Univariable Result	Multivariable Result	Reference
**Social determinants of health**						
	Domestically [b] acquired: semi-skilled manual workers	*jejuni*	Fluoroquinolones	OR = 1.71 (0.96-3.04), p=0.06	No analysis [a]	CSSSC [c], et al, 2002
	Domestically acquired infection 1: student	Not specified	Fluoroquinolones	OR = 0.0 (0.0-0.7), p=0.01	No analysis [a]	Evans M, et al, 2009
	Sex: male vs other	Not specified	Fluoroquinolones	p=0.55	No analysis	Nelson J, et al, 2004
	Sex (female)	*jejuni*	Fluoroquinolones	OR = 0.92 (0.32-2.68), p=0.88	No analysis [a]	Cha W, et al, 2016
	Gender [male vs other]	Not specified	Fluoroquinolones	OR = 0.9 (0.6-1.4), p=0.71	No analysis [a]	Evans M, et al, 2009
	Sex: female vs male	*jejuni*, *coli*, other	Fluoroquinolones	P=0.05 (no OR given)	No analysis [a]	Johnson J, et al, 2008
	Gender/sex [male vs. not male]	*jejuni*, *coli*	Fluoroquinolones<br>Tetracyclines	No analysis	No analysis	Moore J, et al, 2002
	Gender [male vs female]	*jejuni*	Fluoroquinolones<br>Macrolides	No analysis	No analysis	Ghunaim H, et al, 2015
	Race: Asian vs other	*jejuni*	Not specified	OR = 2.3 (1.4-3.9)	No analysis	Patrick M, et al, 2018
	Race: white vs other	Not specified	Fluoroquinolones	No analysis	No analysis	Nelson J, et al, 2004

Category	Factor	Species	AMR outcome	Univariable Result	Multivariable Result	Reference
**Individual characteristics**						
	Age (years)	*jejuni*	Fluoroquinolones	No analysis	OR = 1.05 (0.99-1.1), p=0.0536	Cha W, et al, 2016
	Age, 4 categories = <28, 28-37, 38-49, 50+	*jejuni*, *coli*, other	Fluoroquinolones	p=0.03 (no OR given)<br>28-37yrs vs <28 yrs: OR = 0.2 (0.04-0.8)<br>38-49 yrs vs <28 yrs: OR = 1.0 (0.3-4.1)<br>≥50 yrs vs <28 yrs: OR = 0.2 (0.04-0.9)	p=0.03 overall	Johnson J, et al, 2008
	Age class [Age categories: <1, 1, 2, 3-12, 19-75)	*jejuni*	Fluoroquinolones<br>Macrolides	No analysis	No analysis	Ghunaim H, et al, 2015
	Age group by class of drug [Age categories: 0-9, 10-19, 20-29, 30-39, 40-49, 50-59, 60-69, 70-79, 80+)	Not specified	Fluoroquinolones<br>Macrolides	No analysis	No analysis	Koningstein M, et al, 2011
	Age group of 20-64 compared to the age group of 0-6	*jejuni*, *coli*	Fluoroquinolones<br>Macrolides	No analysis	No analysis	Uzunovic-Kamberovic S, et al, 2009
	Age: over vs under 20	*jejuni*	Not specified	OR = 1.4 (1.1-1.8)	No analysis	Patrick M, et al, 2018

Category	Factor	Species	AMR outcome	Univariable Result	Multivariable Result	Reference
**Individual characteristics**						
	Age group 1= 18-44 vs <18 years	Not specified	Fluoroquinolones	OR = 2.8 (1.1-7.7), p=0.03	OR = 1.5 (0.5-4.0), p=0.47	Evans M, et al, 2009
	Age group 2 = 45-64 vs <18	Not specified	Fluoroquinolones	OR = 4.3 (1.8-11.6), p=0.004	OR = 2.3 (0.9-6.2), p=0.09	Evans M, et al, 2009
	Age group 3: 65+ vs <18	Not specified	Fluoroquinolones	OR = 2.8 (1.1-8.1), p=0.04	OR = 2.0 (1.0-8.2), p=0.04	Evans M, et al, 2009
	Domestically [b] acquired: school children	*jejuni*	Fluoroquinolones	OR = 0.47 (0.22-1.03), p=0.05	No analysis [a]	CSSSC [c], et al, 2002
	Living with a child under 5 years (versus not)	Not specified	Fluoroquinolones	OR = 0.3 (0.2-0.7), p=0.004	No analysis [a]	Evans M, et al, 2009
	Domestically acquired infection 2: living with child under 5 years	Not specified	Fluoroquinolones	OR = 0.4 (0.1-1.0), p=0.05	No analysis [a]	Evans M, et al, 2009
	Domestically [b] acquired: retired individuals	*jejuni*	Fluoroquinolones	OR = 1.32 (0.96-1.80), p=0.08	No analysis [a]	CSSSC [c], et al, 2002
	Men who have sex with men (MSM)	*jejuni*	Fluoroquinolones<br>Tetracyclines<br>Macrolides	No analysis	No analysis	Gaudreau C, et al, 2003
	MSM	*jejuni*	Fluoroquinolones	No analysis	No analysis	Gaudreau C, et al, 2015

Category	Factor	Species	AMR outcome	Univariable Result	Multivariable Result	Reference
			Quinolones			
			Tetracyclines			
**Season**						
	Season (winter)	*jejuni*	Fluoroquinolones	OR = 3.27 (0.92-11.58), p=0.056	OR = 8.1 (0.9-72.7), p=0.0614	Cha W, et al, 2016
	Domestically [b] acquired: winter (versus other seasons)	*jejuni*	Fluoroquinolones	OR = 1.67 (1.24-2.26), p=0.0007	No analysis [a]	CSSSC [c], et al, 2002
	Domestically [b] acquired: autumn (versus other seasons)	*jejuni*	Fluoroquinolones	OR = 1.60 (1.21-2.12), p=0.0008	No analysis [a]	CSSSC [c], et al, 2002
	Domestically [b] acquired: summer (versus other seasons)	*jejuni*	Fluoroquinolones	OR = 0.44 (0.32-0.60), p<0.001	OR = 0.46 (0.33-0.65), p<0.001	CSSSC [c], et al, 2002
	Season of reported infection (summer, autumn, winter, spring)	*jejuni*, *coli*, other	Fluoroquinolones	p<0.001	No analysis [a]	Johnson, J et al, 2008
**Miscellaneous Characteristics**						
	Difference between two study periods: 1995-1997 (comparison group) compared to 1998-2000 (exposed group)	*jejuni*	Fluoroquinolones	No analysis	No analysis	Hakanen, et al, 2003
	Species: C.jejuni	*jejuni*, *coli*, other	Fluoroquinolones	p=0.03	No analysis [a]	Johnson J, et al, 2008

Category	Factor	Species	AMR outcome	Univariable Result	Multivariable Result	Reference
	Campylobacter species	*jejuni, coli*	Quinolones<br>Macrolides	No analysis	No analysis	Patrick M, et al, 2018

[a] Not included in final model

[b] The term 'Indigenous' is in the source paper, but is synonymous with and meant to represent domestic acquisition of infection (i.e., not obtained during travel).

[c] CSSSC = *Campylobacter* Sentinel Surveillance Scheme Collaborators.

* Per the scoping review protocol, data were only extracted if results compared infections with resistant strains to those with susceptible strains. Data include the estimate of the measure of association from the model (OR = odds ratio), the 95% confidence interval in brackets, and the p-value, where applicable/provided.
